# Solid State Phase Equilibria of an Al–Sn–Y Ternary System

**DOI:** 10.3390/ma12030444

**Published:** 2019-01-31

**Authors:** Wenchao Yang, Moumiao Liu, Junli Feng, Jingwu Wu, Jun Mao, Zaixiang Du, Xiaojun Ke, Xinjiang Zhang, Yongzhong Zhan

**Affiliations:** 1 School of Materials Science and Engineering, South China University of Technology, Guangzhou 510641, China; ywch053@163.com; 2 Guangxi Key Laboratory of Processing for Non-ferrous Metals and Featured Materials, Nanning 530004, China; liumoumiao0524@163.com (M.L.); maojun960621@163.com (J.M.); imzx_to@126.com (Z.D.); kexiaojun@gxu.edu.cn (X.K.); zhangxinjiang1983@163.com (X.Z.); 3 Shenzhen Exit Inspection and Quarantine Bureau Industrial Products Inspection Technology Center, Shenzhen 518067, China; fjlhhp@foxmail.com (J.F.); wujingw@163.com (J.W.); 4 College of Resources, Environment and Materials, Guangxi University, Nanning 530004, China

**Keywords:** Al–Sn–Y ternary system, Al_3_Y_5_ phase, phase equilibrium

## Abstract

A complete understanding of the solid-state phase equilibria of the ternary Al–Sn–Y system is essential for the development of both Al-based structural materials and Sn-based lead-free solders. In this work, the phase relationships in the Al–Sn–Y ternary system at 473 K were investigated mainly by means of X-ray powder diffraction (XRD), differential scanning calorimetry (DSC) and scanning electron microscopy (SEM) with energy disperse spectroscopy (EDS) analysis. The existence of 12 binary compounds, namely Sn_3_Y, Sn_5_Y_2_, Sn_2_Y, Sn_10_Y_11_, Sn_4_Y_5_, Sn_3_Y_5_, AlY_2_, Al_3_Y_5_, Al_2_Y_3_, AlY, Al_2_Y and α–Al_3_Y, was confirmed. Controversial phases (Sn_5_Y_2_ and Al_3_Y_5_) were found in this work. This isothermal section consisted of 15 single-phase regions, 27 two-phase regions and 13 three-phase regions. No ternary compounds were found and none of the other phases in this system revealed a remarkable solid solution at 473 K.

## 1. Introduction

Al–based alloys, which consist of Al–Pb and Al–Sn, are widely used for sliding bearing applications due to their good load carrying capacity, fatigue resistance, wear resistance and sliding properties [1,2,3]. However, because of toxic Pb, environmental legislation has driven manufacturers to eliminate Pb from bearing alloys. Thus, the focus has been concentrated on Al–Sn alloys. Al–Sn based alloys are simple eutectic binary alloy systems with solid solutions of a wide range of compositions and are well known as soft tribological alloys [3]. However, the main challenges of Al–Sn based alloys are that the strength of alloys is generally low and can easily form a near-continuous large Sn zone that weakens the interface bonding [4]. Abundant attempts, such as alloying addition, to improve preparation methods have been made to overcome those drawbacks. Al–Sn–Si [5], Al–Sn–Bi [6] and Al–Sn–Mg [4] alloys have been researched with the aim of enhancing the strength of Al-based bearing alloys. It is well known that the addition of small amounts of rare earth elements can improve the microstructures and properties of aluminum alloys [7,8,9]. Meanwhile, Sn–Al eutectic alloy has the potential to be a new system of lead-free solder because it is similar to existing systems, such as the Sn–Zn system and the Sn–Cu system. Rare earth (RE) is an important kind of alloying additive for metallic materials which can significantly improve the properties of alloys by affecting microstructure and refining grain. The ternary Al–Sn–Y system [10] has been reported before but it is only part of the section (65 at.% Y or less) at room temperature, which is not enough for the application of alloys at high temperatures. 

Therefore, a complete knowledge of the phase diagram of the ternary Al–Sn–Y system is essential for a better understanding of this system. The work presented in this article aims to determine the Al–Sn–Y phase equilibrium at 473 K. It is expected that this study will give further insights into the Al–Sn–Y ternary system for practical applications.

## 2. Materials and Methods 

**Figure 1:**
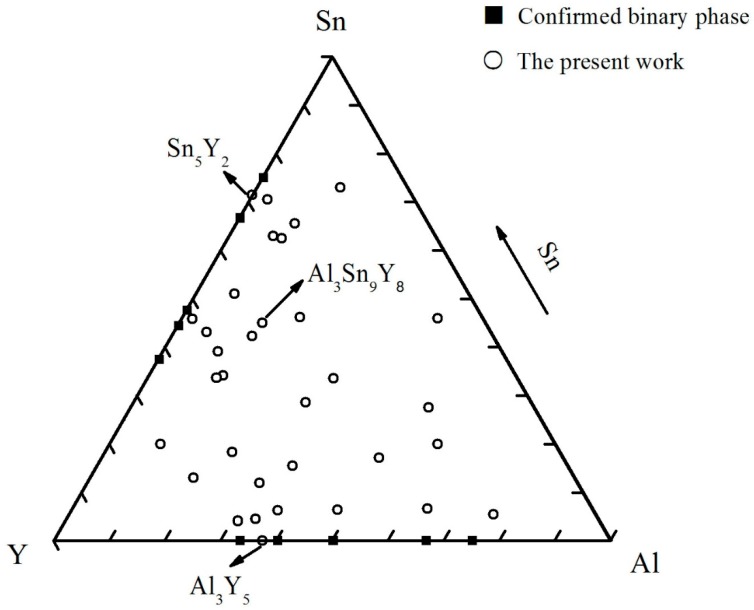
The nominal alloy compositions for the Al–Sn–Y ternary system.

Aluminum (99.9 wt.%), tin (99.9 wt.%) and yttrium (99.99 wt.%) were prepared as raw materials. The alloy compositions of all the samples are plotted in Figure 1. Some components were repeatedly designed for the ideal results. The samples (each 1.5 g) were prepared in an electric arc furnace under an argon atmosphere and a water-cooled copper crucible. In order to obtain a homogeneous composition, each sample was melted three times. For most alloys, the weight loss was generally less than 1 wt.% after being melted. All the samples were sealed in an evacuated quartz tube for homogenization treatment. The alloys which contained more than 50 at.% Sn were homogenized at 673 K for 20 days. Then, the alloys were cooled down to 473 K and maintained for 30 days. Others were kept at 873 K for 10 days and then cooled slowly to 473 K and maintained for 30 days. Finally, all the samples were quenched with ice-water.

All of the homogenized samples were ground into powder and then measured with the help of a Rigaku D/Max 2500V diffractometer (Rigaku, Tokyo, Japan) with CuKα radiation and a graphite monochromator operated at 40 kV, 200 mA. The microstructures and phase analyses were determined by scanning electron microscopy (SEM, Hitachi, Tokyo, Japan) with energy disperse spectroscopy (EDS, Hitachi, Tokyo, Japan) analysis. The temperature of the phase transition was determined using a differential scanning calorimeter (Netzsch, Bavaria, Germany), which was performed in an aluminum crucible with a flowing argon atmosphere as a reference substance between room temperature and 1373 K. The heating and cooling rate used was 10 K·min^−1^.

## 3. Results

### 3.1. Sn–Y Binary System

For the Sn–Y binary system, the existence of the five phases, i.e., Sn_3_Y, Sn_2_Y, Sn_10_Y_11_, Sn_4_Y_5_ and Sn_3_Y_5_, are accepted without question. However, the existence of the phase Sn_5_Y_2_ is controversial. In the Sn–Y phase diagram revised by Okamoto [11], the phase Sn_5_Y_2_ was discovered at the range of temperature from 273 K to 798 K and the structure of the Sn_5_Y_2_ phase was reported in detail, which is in good agreement with the findings of Tang et al. [12]. In that work, the Sn–Y system was investigated by thermodynamic modeling. The Sn_5_Y_2_ phase was considered to have the same structure as the Ge_5_Er_2_ phase, and the lattice parameters were 0.4322 nm (a), 0.4409 nm (b) and 1.9089 nm (c). But the existence of the Sn_5_Y_2_ phase was questioned by Mudryk et al. [13]. When they investigated the R–Fe–Sn ternary systems (R–Y, Gd) at 670 K, Chen et al. [10] and Zhan et al. [14] also reported the same results that phase Sn_5_Y_2_ was not found. However, Romaka et al. [15] later confirmed the existence of the Sn_5_Y_2_ phase in the Sn–Ni–Y ternary system at 670 K. 

**Figure 2:**
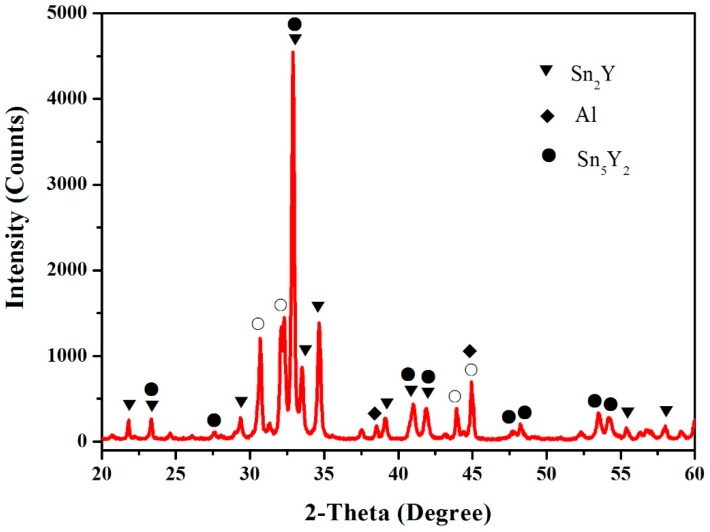
The X-ray powder diffraction (XRD) pattern of the sample (60.5 at.% Sn, 12.5 at.% Al and 27 at.% Y). The symbol ○ is used to indicate Sn.

**Figure 3:**
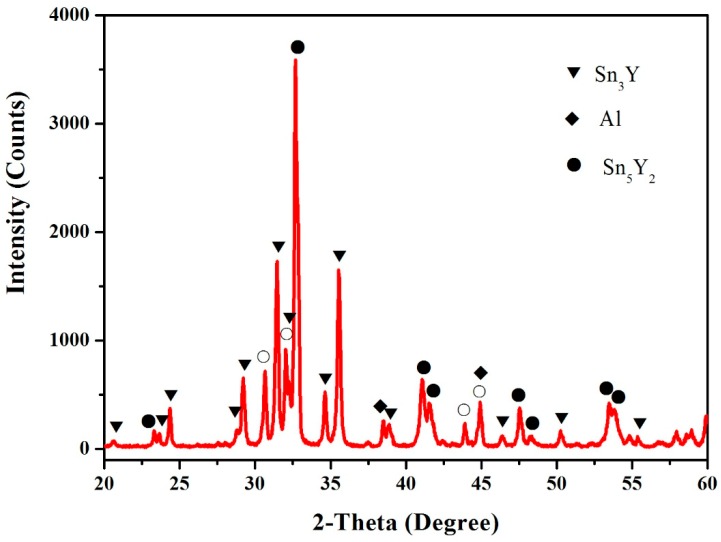
The XRD pattern of the sample (64.5 at.% Sn, 12.5 at.% Al, 23 at.% Y). The symbol ○ is used to indicate Sn.

In this work, the samples (60.5 at.% Sn, 12.5 at.% Al, 27 at.% Y, 64.5 at.% Sn, 12.5 at.% Al and 23 at.% Y) were prepared to verify the existence of the Sn_5_Y_2_ phase. Figure 2 shows the X-ray powder diffraction (XRD) pattern of the sample (60.5 at.% Sn, 12.5 at.% Al and 27 at.% Y), which illustrates the existence of Sn_2_Y, Sn_5_Y_2_ and Al. Figure 3 shows the pattern prepared with the atomic proportion of 64.5 at.% Sn, 12.5 at.% Al and 23 at.% Y. It indicates the existence of the three phases—Sn_3_Y, Sn_5_Y_2_ and Al. In Figure 2 and Figure 3, the Sn phase was found. A possible reason for this is that when the Sn content is higher, it is easy to separate out tin whiskers during annealing over a longer time [16,17]. However, the XRD patterns of the samples clearly showed the existence of the Sn_5_Y_2_ phase, which was confirmed in the Al–Sn–Y ternary system at this investigated temperature.

### 3.2. Al–Y Binary System

For the Al–Y binary system, the existence of the five phases, i.e., Y_2_Al, Y_3_Al_2_, YAl, YAl_2_ and YAl_3_, are accepted without question. Bailey [18] reported early on that two structurally related polymorphic forms of Al_3_Y have been corroborated—a low temperature form with the hexagonal Ni_3_Sn-type structure (α–YAl_3_) and a high temperature form with a rhombohedra BaPb_3_-type structure (β–YAl_3_). At the temperature of this work, the YAl_3_ phase is α–YAl_3_. The Al–Y binary system was also investigated thermodynamically by Lukas [19]. The Y_5_Al_3_ phase was found by Lukas and the structure of Y_5_Al_3_ phase was identified by Richter et al. [20]. Liu et al. [21] also experimentally investigated the Al–Y phase diagram and failed to confirm the existence of the Y_5_Al_3_ phase. After that, in the studies of many ternary systems, such as Al–Fe–Y [22], Al–Zr–Y [23], Al–Sb–Y [24] and Al–Sn–Y [10], the Y_5_Al_3_ phase was not found. However, Liu et al. [25] thermodynamically assessed the Al–Zn–Y system and found that the binary compound Y_5_Al_3_ forms through the reaction L + Y_3_Al_2_ ⇔ Y_5_Al_3_ + YZnAl at 997 K. However, the temperature range of Y_5_Al_3_ has not been clearly identified.

**Figure 4:**
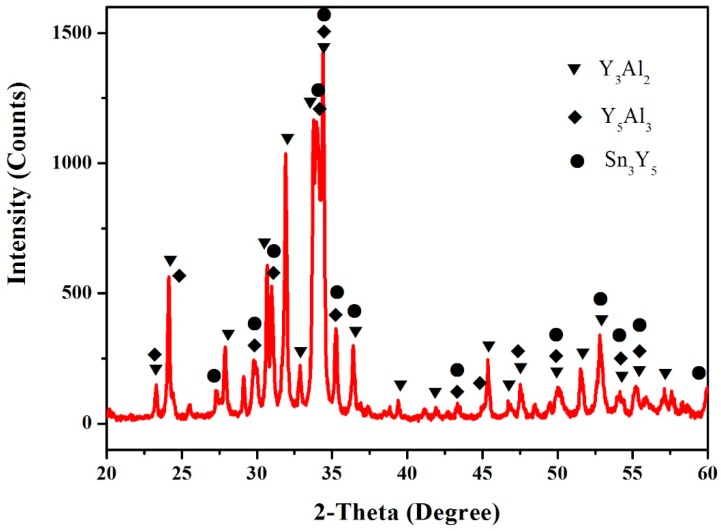
The XRD pattern of the sample (4 at.% Sn, 35 at.% Al and 61 at.% Y).

**Figure 5:**
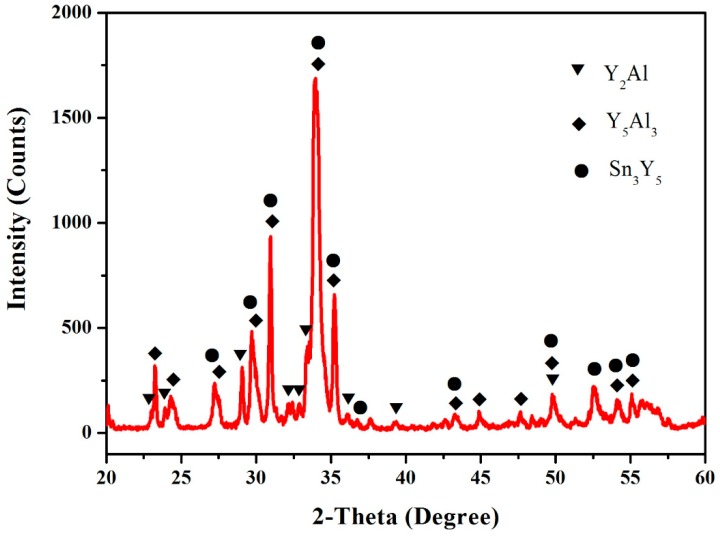
The XRD pattern of the sample (5 at.% Sn, 30 at.% Al, 65 at.% Y).

In order to obtain a believable result, the samples (4 at.% Sn, 35 at.% Al, 61 at.% Y, 5 at.% Sn, 30 at.% Al and 65 at.% Y) were prepared. Figure 4 shows that the XRD pattern of the sample (4 at.% Sn, 35 at.% Al and 61 at.% Y) illustrates the existence of Y_3_Al_2_, Y_5_Al_3_ and Sn_3_Y_5_. The XRD pattern of the sample (5 at.% Sn, 30 at.% Al, 65 at.% Y) illustrates the existence of Y_2_Al, Y_5_Al_3_ and Sn_3_Y_5_, as shown in Figure 5, which indicates the existence of the Y_5_Al_3_ phase.

### 3.3. Sn–Al Binary System

**Figure 6:**
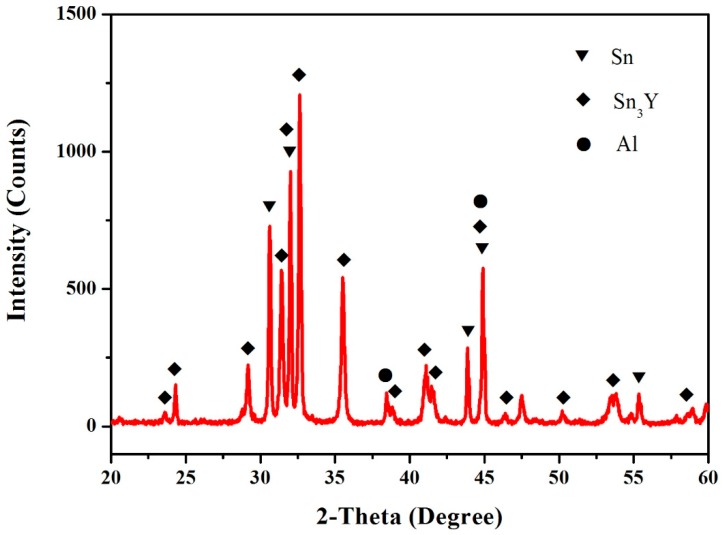
The XRD pattern of the sample (69.8 at.% Sn, 14.9 at.% Al, 15.3 at.% Y).

There was no compound found in the Sn–Al system. Figure 6 shows that the XRD pattern of the sample (69.8 at.% Sn, 14.9 at.% Al, 15.3 at.% Y) illustrates the existence of Sn, Al and Sn_3_Y. The crystal structure data of the intermetallic compounds in the Sn–Y, Al–Y and Sn–Al binary systems at 473 K are given in Table 1.

### 3.4. Al–Sn–Y Ternary System

**Figure 7:**
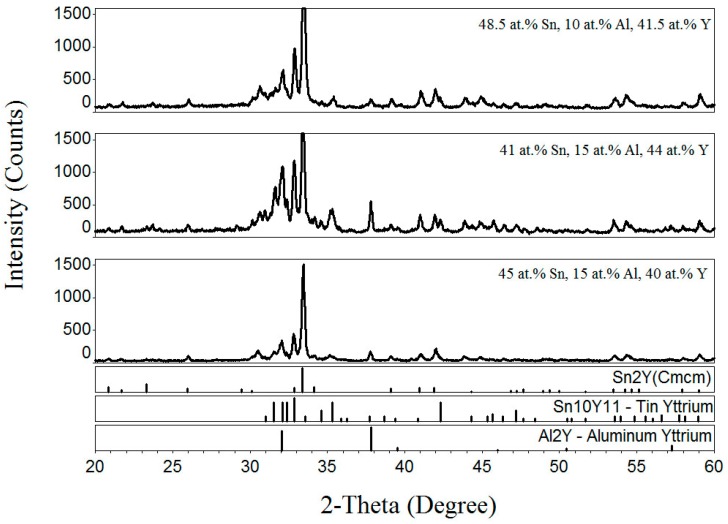
The XRD patterns of the samples (48.5 at.% Sn, 10 at.% Al, 41.5 at.% Y, 41 at.% Sn, 15 at.% Al, 44 at.% Y and 45 at.% Sn, 15 at.% Al, 40 at.% Y).

For the Al–Sn–Y ternary system, the Al_3_Sn_9_Y_8_ ternary compound was detected by Chen et al. [10] at room temperature. In order to verify the existence of the Al_3_Sn_9_Y_8_ phase at 473 K, the samples (48.5 at.% Sn, 10 at.% Al, 41.5 at.% Y; 41 at.% Sn, 15 at.% Al, 44 at.% Y; 45 at.% Sn, 15 at.% Al, 40 at.% Y) were prepared. The XRD patterns of the samples clearly indicated the existence Sn_2_Y, Sn_10_Y_11_ and YAl_2_, as shown in Figure 7. Thus, the Al_3_Sn_9_Y_8_ ternary compound was not detected in this work.

### 3.5. Isothermal Section

**Figure 8:**
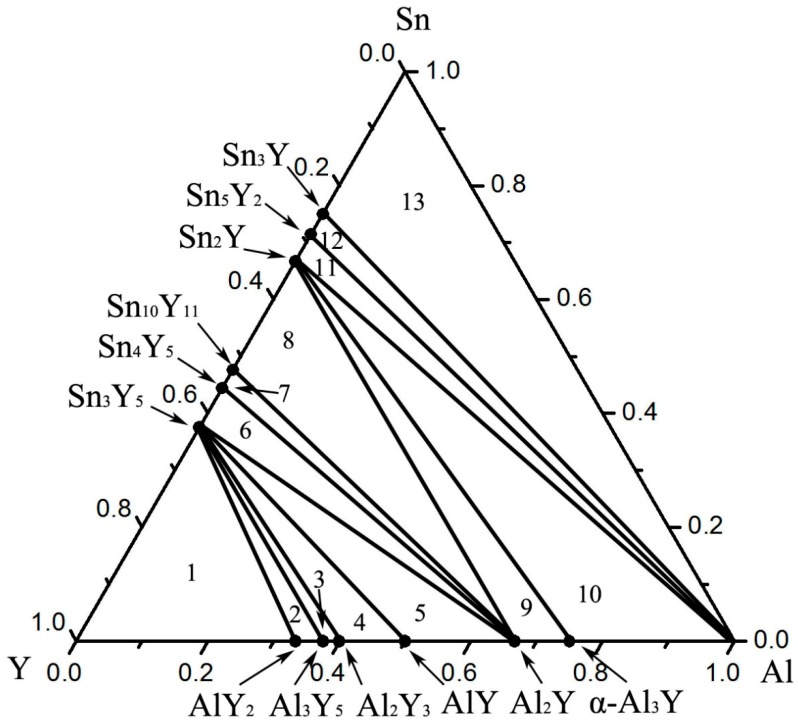
The isothermal section of the Sn–Al–Y ternary system at 473 K.

**Figure 9:**
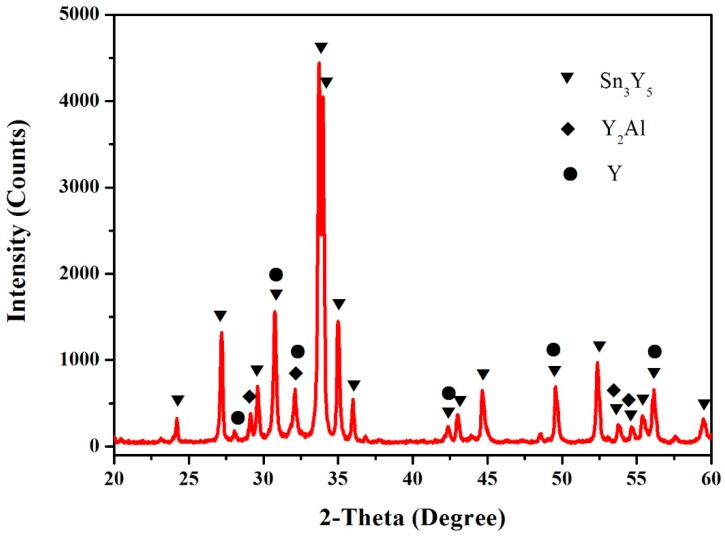
The XRD pattern of the sample (20 at.% Sn, 9 at.% Al and 71 at.% Y).

**Figure 10:**
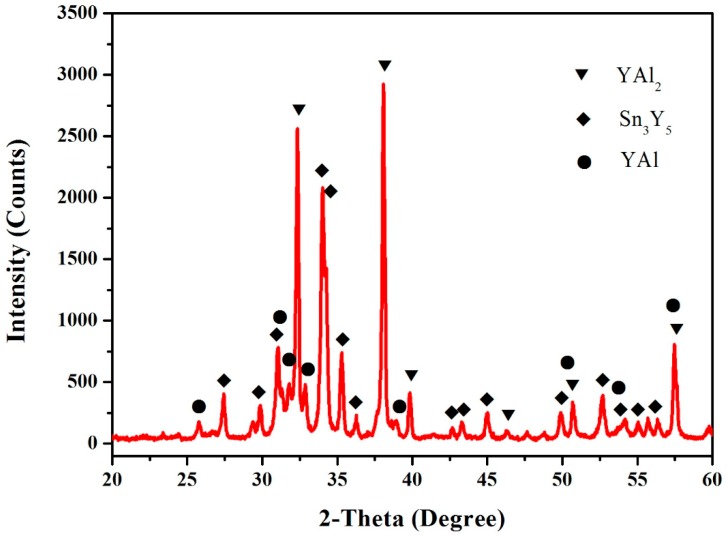
The XRD pattern of the sample (6.5 at.% Sn, 47.8 at.% Al and 45.7 at.% Y).

**Figure 11:**
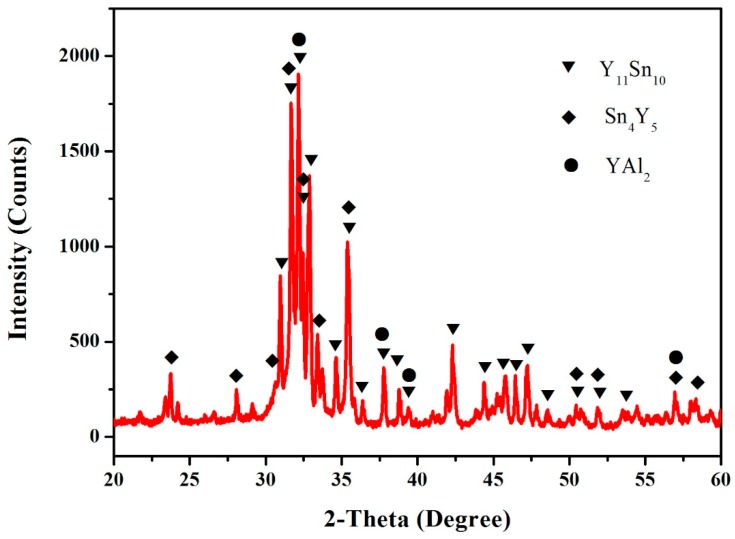
The XRD pattern of the sample (39 at.% Sn, 10 at.% Al and 51 at.% Y).

**Figure 12:**
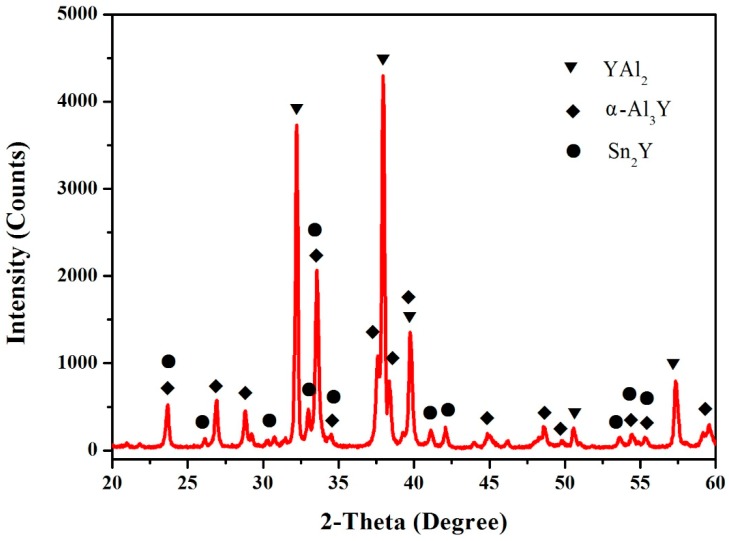
The XRD pattern of the sample (6.5 at.% Sn, 64 at.% Al and 29.5 at.% Y).

**Figure 13:**
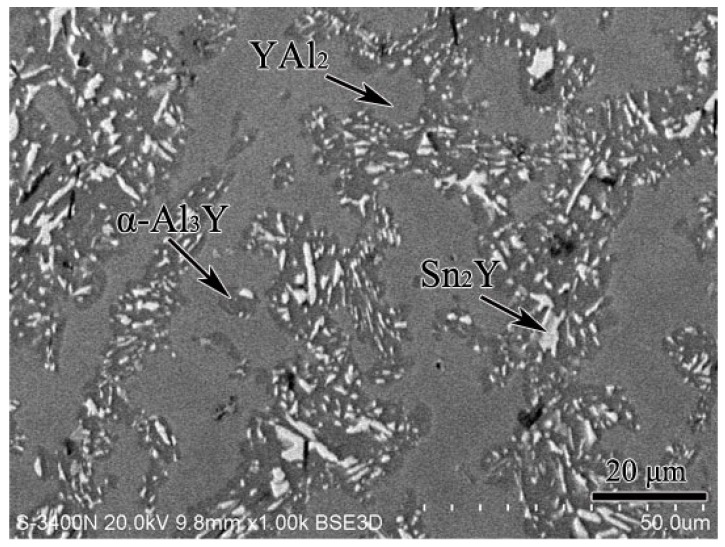
The scanning electron microscopy (SEM) micrograph of the equilibrated alloy 6.5 at.% Sn, 64 at.% Al, 29.5 at.% Y illustrating the existence of YAl_2_, Sn_2_Y and α–Al_3_Y.

**Figure 14:**
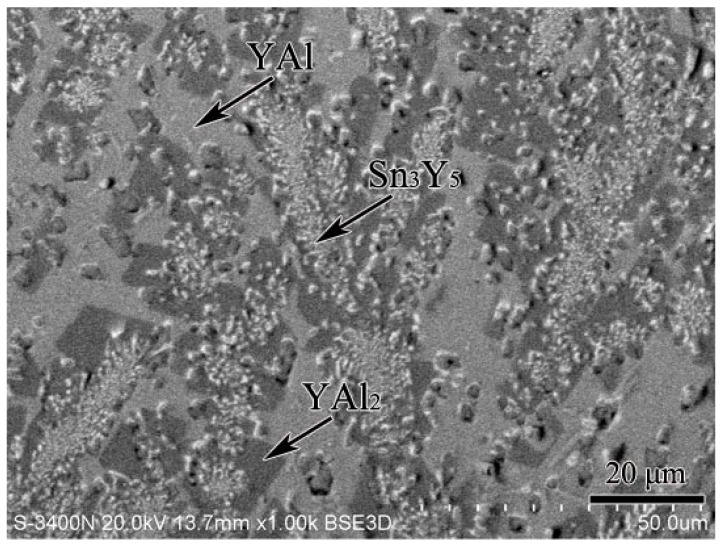
The SEM micrograph of the equilibrated alloy 6.5 at.% Sn, 47.8 at.% Al, 45.7 at.% Y illustrating the existence of YAl, YAl_2_ and Sn_3_Y_5_.

**Figure 15:**
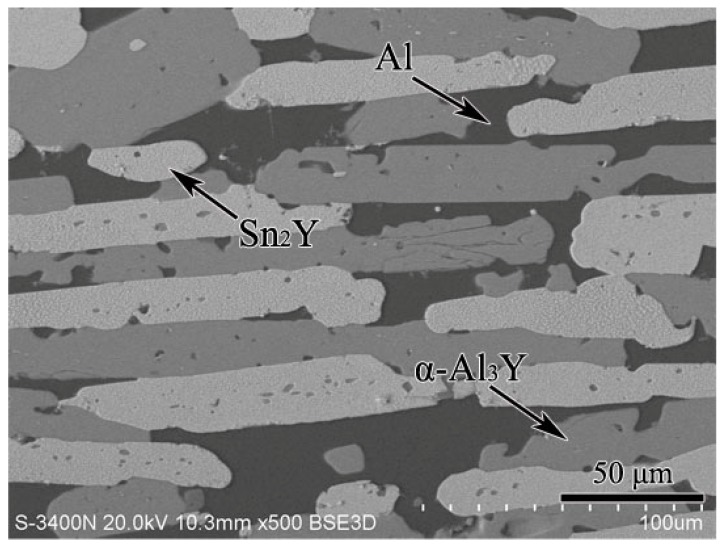
The SEM micrograph of the equilibrated alloy 20 at.% Sn, 59 at.% Al, 21 at.% Y illustrating the existence of Al, Sn_2_Y and α–Al_3_Y.

According to the XRD, SEM/EDS and differential scanning calorimeter (DSC) analysis, the isothermal section of the Al–Sn–Y ternary system at 473 K is shown in Figure 8. This isothermal section consists of 15 single phase regions, 27 binary phase regions and 13 ternary phase regions. No ternary compounds were found and none of the other phases in this system revealed a remarkable homogeneity range at 473 K. Figure 9 shows the XRD pattern of the sample (20 at.% Sn, 9 at.% Al, 71 at.% Y) indicating the existence of Sn_3_Y_5_, Y_2_Al and Y. The XRD pattern of the equilibrated sample with a stoichiometric composition of 6.5 at.% Sn, 47.8 at.% Al, 45.7 at.% Y indicated the existence of Sn_3_Y_5_, Al_2_Y and YAl, as shown in Figure 10. In addition, Figure 11 shows the XRD pattern of the sample (39 at.% Sn, 10 at.% Al, 51 at.% Y) indicating the existence of Sn_4_Y_5_, Al_2_Y and Sn_10_Y_11_. Figure 12 shows the XRD pattern of the sample (6.5 at.% Sn, 64 at.% Al, 29.5 at.% Y) indicating the existence of α–Al_3_Y, Al_2_Y and Sn_2_Y. The XRD results confirm that nine binary compounds, namely Sn_3_Y, Sn_2_Y, Sn_10_Y_11_, Sn_4_Y_5_, Sn_3_Y_5_, Y_2_Al, Y_3_Al_2_, YAl, YAl_2_ and α–YAl_3_, exist in this system at 473 K. The SEM photographs (as shown in Figure 11, Figure 12, Figure 13, Figure 14 and Figure 15) also clearly display the existence of some phases (identified by EDS). Constitutions of the ternary phase regions and compositions of the typical alloys are given in Table 2.

## 4. Conclusions

The isothermal section of the Sn–Al–Y ternary system at 473 K was experimentally constructed in this work. This isothermal section consists of 15 single-phase regions, 27 two-phase regions and 13 three-phase regions. The existence of 12 binary compounds was confirmed; namely, Sn_3_Y, Sn_5_Y_2_, Sn_2_Y, Sn_10_Y_11_, Sn_4_Y_5_, Sn_3_Y_5_, AlY_2_, Al_3_Y_5_, Al_2_Y_3_, AlY, Al_2_Y and α–Al_3_Y. No ternary compound was found. 

## Figures and Tables

**Table 1:** Binary crystal structure data of the Al–Sn–Y system at 473 K.

Phase	Pearson’s Symbol	Crystal Structure	Space Group	Lattice Parameters (nm)			Refs.
				a	b	c	
Sn_3_Y	oC16	Gd_4_Sn_11_	Amm2	0.4345	0.4391	2.1937	[12]
Sn_5_Y_2_	oP14	Ge_5_Er_2_	Pmmm	0.4322	0.4409	1.9089	[12]
Sn_2_Y	oC12	Si2Zr	Cmcm	0.4398	1.632	0.4304	[12]
Sn_10_Y_11_	tI84	Ge_10_Ho_11_	I4/mmm	1.154	–	1.692	[12]
Sn_4_Y_5_	oP36	Ge_4_Sm_5_	Pnma	0.805	1.529	0.805	[12]
Sn_3_Y_5_	hP16	Si_3_Mn_5_	P6_3_/mcm	0.8902	–	0.6536	[12]
αYAl_3_	hP8	Ni_3_Sn	P6_3_/mmc	0.6276	–	0.4582	[26]
YAl_2_	cF24	Cu_2_Mg	$Fd\bar{3}m$	0.78611	–	–	[26]
YAl	oC8	CrB	Cmcm	0.3884	1.1522	0.4385	[26]
Y_3_Al_2_	tP20	Al_2_Zr_3_	P4_2_/mnm	0.8239	–	0.7648	[26]
Y_2_Al	oP12	Co_2_Si	Pnma	0.6642	0.5084	0.9469	[26]
Y_5_Al_3_	hP16	Mn_5_Si_3_	P6_3_/mcm	0.8787	–	0.6435	[20]

**Table 2:** Details of the phase regions and typical samples in the Al–Sn–Y system at 473 K.

Phase Regions	Alloy Composition (at.%)			Phase Composition
	Sn	Al	Y	
1	20	9	71	Y + Sn_3_Y_5_ + Y_2_Al
2	5	30	65	Y_2_Al + Sn_3_Y_5_ + Y_5_Al_3_
3	4	35	61	Y_5_Al_3_ + Sn_3_Y_5_ + Y_3_Al_2_
4	6.3	37	56.7	Y_3_Al_2_ + Sn_3_Y_5_ + AlY
5	6.5	47.8	45.7	AlY + Sn_3_Y_5_ + Al_2_Y
6	33.5	12.5	54	Al_2_Y + Sn_3_Y_5_ + Sn_4_Y_5_
7	39	10	51	Sn_4_Y_5_ + Al_2_Y + Sn_10_Y_11_
8	45	15	40	Sn_10_Y_11_ + Al_2_Y + Sn_2_Y
9	6.5	64	29.5	Sn_2_Y + Al_2_Y + α–Al_3_Y
10	20	59	21	α–Al_3_Y + Sn_2_Y + Al
11	60.5	12.5	27	Al + Sn_2_Y + Sn_5_Y_2_
12	64.5	12.5	23	Sn_5_Y_2_ + Al + Sn_3_Y
13	69.8	14.9	15.3	Sn_3_Y + Al + Sn
